# Managing Everyday Life in Double Exposure: Frail Older People’s Experiences During a Pandemic

**DOI:** 10.1007/s44474-026-00001-7

**Published:** 2026-07-10

**Authors:** Katharina Sjöberg, Synneve Dahlin-Ivanoff, Katarina Wilhelmson, Isabelle Andersson Hammar

**Affiliations:** 1 Department of Psychiatry and Neurochemistry, Institute of Neuroscience and Physiology, The Sahlgrenska Academy, University of Gothenburg, Gothenburg, Sweden; 2 Department of Acute Medicine and Geriatrics, Sahlgrenska University Hospital, Gothenburg, Sweden; 3 Department of Health and Rehabilitation, Institute of Neuroscience and Physiology, The Sahlgrenska Academy, University of Gothenburg, Gothenburg, Sweden

**Keywords:** Activities of daily living, Frailty, Leisure activities, Pandemics, Qualitative research, Social isolation

## Abstract

**Introduction:**

Research has shown that older people experienced mental and physical health issues due to isolation during the Covid-19 pandemic. However, more in-depth knowledge is needed regarding the consequences for frail older people.

**Aim:**

To explore frail older people’s experiences of managing everyday life in quarantine during the first year of the Covid-19 pandemic.

**Material and Methods:**

Twenty people were interviewed, and the data were analyzed using qualitative content analysis.

**Results:**

The people experienced *powerlessness under the restrictions* and *limitations in everyday life beyond control.* To cope with these experiences, they *accepted and trusted the restrictions* and *strived for meaningfulness in everyday life.*

**Conclusions:**

Frail older people experienced managing everyday life in double exposure during the pandemic, where personal vulnerabilities and external restrictions intersected, limiting their ability to adapt and maintain autonomy in everyday life. Supporting meaningful routines and enabling participation are therefore central, underscoring the important role of occupational therapy in mitigating the impact of disrupted everyday life.

**Significance:**

These findings contribute to a more nuanced understanding of the needs of frail older people. This approach is essential to sustaining leisure activities, fostering social connections, and promoting health and well-being during future disruptive events.

## Introduction

Beginning in March 2020, people aged 70 and older in Sweden were encouraged to restrict their social contacts and avoid gatherings because of the Covid-19 pandemic [1]. The pandemic imposed a significant burden on healthcare, and prioritizations were required to care for all Covid-19 patients. A policy report [2] revealed deficiencies in coordination between different healthcare providers, which could particularly affect people with more extensive care needs [2]. Frail older people constitute one such group. Frailty refers to older people’s difficulty in managing daily life due to reduced bodily functions, increased vulnerability, and a diminished ability to handle various stressors, which can result in negative health outcomes [3]. Physical frailty can be described using the phenotype model [4], where at least three of the following criteria must be met for a person to be considered frail: general weakness, reduced endurance, weight loss, low physical activity, and slow walking speed [4]. Frailty can contribute to a reduced quality of life, impaired cognition, and an increased risk of depression and pain. Advanced age increases the risk of frailty [5], and after the age of 80, there is a significant increase [6]. Frailty is not constant and can change over time. It can be treated, among other things, through physical exercise, diet/nutritional supplements, and medication review [6]. Treatment of frailty is proposed to occur through a team-based approach targeting disease prevention and advice on lifestyle factors in a way that supports the autonomy of frail older people [7].

Older people often develop habits within functional contexts, which help sustain their ability to perform daily activities despite changes in personal capacity or environmental conditions [8]. Restrictions and quarantine recommendations for people aged 70 and older during the pandemic resulted in a clear disruption of habits and routines. Continuity can be used as a strategy to compensate for impairments that occur with normal ageing. External continuity involves routines that encompass activities, skills, environments, and social relationships [9]. Prolonged home isolation can negatively affect health, especially among frail older people who experience disruptions in their daily routines [1]. Such interruptions could be stressful and when their personal capacity is reduced, developing new strategies becomes challenging and demanding [10]. Among people aged 85 years and older, quality of life is strongly associated with engagement in daily activities. This is especially true for leisure activities that people perceive as meaningful [11]. There are also associations between low self-rated health and the experience of limited self-determination in older people’s daily activities [12].

Among older people living in nursing homes, social isolation became particularly evident during the pandemic [13]. Although digital technologies were introduced to support social contact [13], many older people lacked access or competence, which further limited their participation in everyday activities [14]. Internationally, people over the age of 70 experienced both mental and physical ill health due to isolation during the pandemic, and more research on the consequences is needed [1]. Even in Sweden, where restrictions were less stringent, many very old people reported negative effects on their mental health, with links to loneliness and difficulties adhering to the public recommendations [15]. Further, even after only a few months of restrictions, older people in Sweden experienced alterations in daily activities, routines and habits [16].

According to the capability approach, life can be understood as different ways of functioning grounded in what people are able to be and do, encompassing aspects such as happiness, good health, and participation in society [17]. These ideas align closely with the core occupational therapy understanding of human occupation. Wilcock and Hocking [18] describe occupation as comprising the interrelated dimensions of doing, being, becoming, and belonging, which together support health, identity, growth, and social connectedness. These dimensions are not separate but mutually reinforcing; meaningful occupation emerges when people can integrate action, reflection, development, and social affiliation in their everyday lives [18]. For frail older people, capability extends beyond objective health and includes the ability to remain engaged in meaningful occupations and pursue personally valued goals. This underscores the importance of creating everyday opportunities for people to express their will, make decisions, and exercise self-determination [19]. Consequently, a person-centred approach grounded in active partnership and shared decision-making becomes essential, ensuring that care and support are tailored to the person’s values, preferences, and occupational goals [20].

To date, there is limited knowledge about how frail older people, the most vulnerable group of older people in Sweden, experienced the changes brought about by the Covid-19 pandemic. This gap is particularly relevant to occupational therapy, which centres on enabling participation and supporting daily routines in the face of functional decline, environmental barriers, or social restrictions. Given that capability and meaningful occupation are central to wellbeing, it is notable that research has not yet fully examined how frail older people navigated disruptions to their everyday lives during the pandemic. Understanding these experiences is essential not only for preparing for future public health crises but also for informing occupational therapy strategies in more ordinary circumstances where older people face prolonged isolation due to, for example, illness, mobility limitations, and restricted social networks. A deeper understanding of these experiences can strengthen person-centred strategies aimed at supporting continuity, self-determination, and participation in daily life in frail older people. To address this gap, the present study explores frail older people’s experiences of managing everyday life in quarantine during the first year of the Covid‑19 pandemic.

## Methods

### Design

The study has a qualitative design using semi-structured telephone interviews to collect the data. A qualitative content analysis was chosen for data analysis. The method aims to identify variations by highlighting differences and similarities expressed in codes, categories and themes generated from the data [21].

### Participants and Setting

The current study was conducted within the framework of the larger randomized controlled CGA Swed study [22], which aimed to evaluate comprehensive geriatric assessment for frail older people within Swedish emergency care. The larger study was conducted from March 2016 until January 2020 and included 155 people 75 years or older, in need of emergency care, and screened as frail [22]. For the present qualitative study, a strategic sample was applied [23]. The aim was to achieve a heterogeneous group of frail older people with a variation of age, illness, functional ability, and dependence in activities of daily living. Based on previous experiences in the research group, participants from the main study were selected if they were judged able to participate in a telephone interview and had not developed cognitive or hearing impairments that could hinder participation. The participants also needed to be able to communicate in Swedish.

The final sample consisted of 20 participants aged 78 to 100 years, including 11 women and 9 men. Two lived in nursing homes, all except one were dependent on home-help services, and seven rated their health as good whereas the remaining participants rated their health as fair or poor (Table 1). For detailed information regarding the CGA Swed study, see the study protocol [22].

**Table 1:** Participants’ characteristics

Number	Gender	Age	Marital status	Housing	Elderly care	Cleaning services	Self-rated health**
1	Female	82	Single	Apartment	No	Yes	Good
2	Male	100	Single	Nursing home	Yes	No	Good
3	Male	86	Married/cohabiting	Private house	Yes	Yes	Fair
4	Female	93	Single	Apartment	Yes	No	Fair
5	Male	84	Single	Apartment	No	No	Fair
6	Female	86	Single	Apartment	Yes	No	Good
7	Male	85	Married/cohabiting	Private house	No	No	Good
8	Female	90	Single	Apartment 65 + *	Yes	No	Fair
9	Female	86	Single	Apartment 65 + *	No	No	Good
10	Female	89	Single	Apartment	Yes	No	Good
11	Female	86	Single	Apartment	Yes	No	Fair
12	Female	85	Single	Nursing home	Yes	No	Fair
13	Male	79	Married/cohabiting	Apartment	No	No	Fair
14	Female	88	Single	Private house	Yes	No	Fair
15	Female	91	Single	Apartment	No	No	Good
16	Female	78	Single	Apartment	No	No	Poor
17	Male	85	Married/cohabiting	Private house	Yes	No	Fair
18	Male	90	Married/cohabiting	Apartment	No	No	Fair
19	Male	96	Single	Apartment	Yes	No	Fair
20	Male	90	Single	Apartment	No	No	Fair

^*^Rental apartment complex targeting tenants above the age of 65

^**^Self-rated health (SRH) was measured with the question: In general, would you say your health is excellent, very good, good, fair, or poor?

A written invitation to participate in a telephone interview was sent to a total of 22 older people at the end of September 2020. They were subsequently contacted by the first author (KS) to provide additional information, inquire about their participation, and schedule an interview if they were interested. The first author (KS), who conducted the interviews, had previously met the participants in person during data collection in the CGA Swed study [22].

One person declined due to medical issues, one person passed away during the period between the invitation being sent and the first phone call, and an additional three could not be reached by phone despite repeated attempts. Of the total 22 individuals approached, 16 agreed to participate. To compensate for the dropout of participants after the first invitation and to achieve a representative sample in relation to gender, an additional four invitations were sent out in January 2021. After the second invitation, all eligible participants who were still alive in the main study were contacted, in accordance with the strategic sampling procedure described above. The first author (KS) contacted them in the same manner as described above, and all agreed to participate. In total, 26 people were approached, of whom 20 agreed to participate and completed the interviews.

### Data Collection

The data was collected through semi-structured interviews, conducted via telephone and recorded on a mobile device. An interview guide was used, beginning with an initial broader open question about how the participants’ lives had been affected during the period of the pandemic, with quarantine and restrictions. This was followed by questions concerning areas of inquiry, covering daily activities, leisure activities/activities performed for pleasure, contacts with relatives and friends, assistance from relatives or elder care services, and contact with healthcare services. Self-rated health (SRH) was measured with the single question: In general, would you say your health is excellent, very good, good, fair, or poor?

Telephone interviews were used due to prevailing social distancing restrictions during the pandemic. It was not ethically justifiable to expose frail older people to the risk of infection that could occur during a personal meeting, even if restrictions were followed. It could also have meant that several participants might have been unwilling to participate out of fear of infection.

An initial pilot interview was conducted to test the interview guide and the role of the interviewer. The results were reviewed and discussed with the rest of the authors, who provided feedback to the first author concerning the follow-up questions and the interview technique. The pilot interview and the subsequent 15 interviews were conducted over a two-month period, from October to early December 2020. In parallel with conducting the interviews, the inclusion process was ongoing, and participants were continuously enrolled. At the same time, the authors listened to the recorded interviews and provided continuous feedback on the interview technique.

During this period, all interviews were also transcribed verbatim by the first author (KS). The supplementary four interviews in the second round were conducted during January and February 2021 and were transcribed in the same way. The interview duration ranged from 11 to 33 min, with a median length of 23 min.

### Data Analysis

**Fig. 1:**
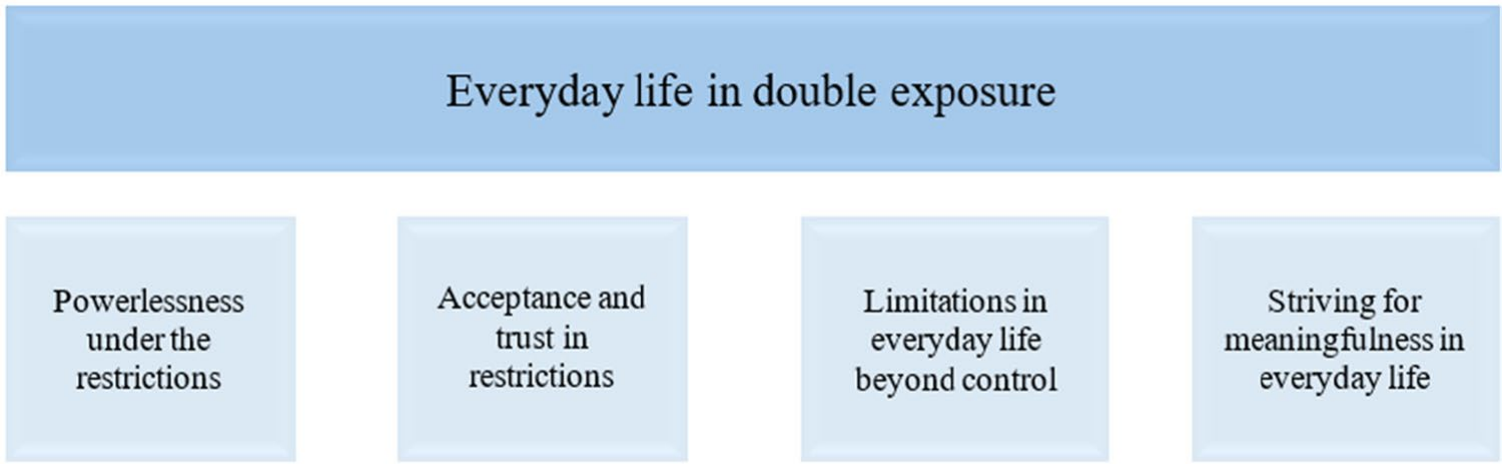
Overview of the main theme and subthemes

The process for qualitative content analysis described by Graneheim and Lundman [21] was used as the basis for the analysis. Data analysis began in parallel with conducting the interviews, listening to them, and transcribing them verbatim. The interviews were analyzed in their entirety; meaning units were identified, and primary codes close to the text were written in the margins of the transcribed interviews. This was followed by further readings with notes on meaning units at a detailed level, which were then compiled into common codes in text documents. Codes were then grouped, and primary categories were created. The analysis then moved back and forth between the transcribed interviews and the coded material; relevant quotes belonging to each category were selected and grouped together. The codes were repeatedly regrouped into categories, from which themes were identified. This process resulted in one main theme and four subthemes (Fig. 1). The first author (KS) was responsible for the analysis with support from the last author (IAH). Throughout the work, the results were also discussed with the other coauthors (SDI and KW).

### Ethics

This study was part of a larger study [22], for which ethical approval was obtained from the Regional Ethics Review Board in Gothenburg (ref. no. 899–15). Frail older people are a vulnerable patient group with multimorbidity and extensive healthcare needs, and they are therefore underrepresented in research. Upon inclusion in the main study, information about the study was provided both verbally and in writing, including its purpose, how it would be conducted, what was expected of the participants, that participation was voluntary, and that the participants could withdrawn at any time. Participants signed a consent form at the time of inclusion in the main study. In the study data, all individuals were coded, and no personal data appeared. Lists containing personal data were accessible only to the research staff and stored in a locked cabinet at the University of Gothenburg. For the current study, additional ethical approval was obtained (ref. no. 2020–03809) from the same ethics review board. Verbal consent to participate in this study was obtained in accordance with this approval. All interviews and quotes were coded to ensure anonymity. Frail older people can easily become fatigued, which could pose a risk in this interview study. The author was attentive to this during the interviews. Flexibility was employed to reschedule interview times if needed, and participants were encouraged to discontinue the interview if they felt fatigued.

## Results

Participants’ prior life circumstances significantly influenced everyday life when living with restrictions during the Covid-19 pandemic. *Everyday life in double exposure* reflects the main theme, implying that even before the pandemic, limitations existed across multiple domains in daily life as a consequence of ageing and illness. Dependence on elder care services differed among the participants and could negatively affect control over daily routines and engagement in everyday life. Regarding leisure activities, most had already adapted to a limited capacity, but participants still varied in their ability. Some were able to join different kinds of group activities in society, while others were limited to solitary activities at home, resulting in reduced social interactions. The loss of friends due to old age further limited social interactions, and for some, relatives became the primary social network. At the same time, reliance on support from relatives in everyday life increased which overall heightened the dependence of the frail older person. The restrictions further exacerbated all of these limitations, and the participants had to manage everyday life in double exposure during the pandemic. *Everyday life in double exposure* affected all four subthemes: *Powerlessness under the restrictions*,* Acceptance and trust in restrictions*, *Limitations in everyday life beyond control*, and *Striving for meaningfulness in everyday life.* The main theme is further described in relation to each subtheme, illustrating participants’ experiences of managing life and the imposed restrictions during the pandemic.

### Powerlessness Under the Restrictions

This subtheme captures the tension between adhering to pandemic restrictions and at the same time struggling with one’s interpretation of the restrictions and their impact on everyday life. Being aware of the need to limit one’s social contacts conflicts with simultaneously being dependent on contact with others to manage everyday life. Restrictions were experienced as profoundly limiting, with the pandemic period often referred to as a ‘lost year’. The constraints heightened the awareness of life’s temporal nature, intensifying the desire to engage in meaningful occupations. Participants felt forced to live unnaturally, with restrictions undermining their sense of making decisions on their own and their occupational identity.

You have to follow the given rules … a new kind of rules about how to live … and it’s terrible … to be forced to function in life in a way that is completely abnormal. You can’t go out, you can’t infect others, and you have to be careful not to get infected yourself … Yes, there are a whole lot of rules to follow. (Participant nr 16)

For those residing in care homes, the limitations were particularly severe, such as during certain periods not being allowed to move in common areas and not being able to meet relatives. Witnessing friends or fellow residents’ contract and succumb to the virus added to the emotional burden.

It was me and two others who were sitting here. You understand- a whole number of people died at the same time… so there were only three of us who ate upstairs, out in the common room…and it was… well, I don´t really need to say what the atmosphere was like after that. It was those of us who were grieving who remained. (Participant nr 2)

The influence of relatives’ opinions further shaped adherence to restrictions, both in terms of permissible social interactions and decisions about what activities the frail older person was allowed to engage in. This external control was perceived as restrictive, reinforcing feelings of limited agency. Thoughts were expressed about somehow being able to influence one’s situation but not knowing how to do so.

Yes, you sit here waiting for something else … it’s probably stupid; really, you should get started with something, but everything is discouraging. Whatever you try, it doesn’t work – You can’t go there, – You can’t do that. (Participant nr 15)

### Acceptance and Trust in Restrictions

A strategy used to manage the participants’ *sense of powerlessness* was to *accept and trust the restrictions*. Demonstrating acceptance and trusting the necessity and effectiveness of the restrictions became a way to cope with the double exposure in everyday life. While feelings of resignation and the difficulty of influencing one’s circumstances were acknowledged, acceptance allowed participants to focus on external factors such as vaccination prospects or seasonal changes. Participants’ strict adherence to the measures, such as avoiding all social contact, provided a sense of security. Others adopted a more balanced approach, integrating precautionary measures such as using masks and sanitizers as well as maintaining physical distance while continuing essential activities. Visits to clinics or other services were deemed safe when the restrictions were followed. Healthcare was still sought when needed, and routine visits continued upon invitation. However, participants reliant on elder care services described variations in adherence to safety measures, such as inconsistent use of protective equipment, which undermined their sense of security.

[Talking about the use of protection among staff] No, they don’t, not when they come here, they don’t … but I keep away from them and sit by myself … I’m glad they’ve started helping me again because I had such a hard time. (Participant nr 10)

Those who already had an exposed life situation before the pandemic with low mood, anxiety, and in some cases thoughts of not wanting to continue living faced amplified challenges. Despite this, compliance with restrictions and trust in their necessity persisted. Some of the participants depending on elderly care cancelled services out of fear of infection and past experiences of staff turnover. Having more extensive care needs limited this opportunity. Over time, pandemic-related social behaviours, such as keeping a distance and not shaking hands or hugging, became routine. However, adherence to restrictions occasionally relaxed, with participants prioritizing physical contact with close relatives over the fear of infection, reflecting a balance between emotional and physical needs.

[Talking about help with shopping]: My son has been very restrictive, and then he just placed it inside the door and left … but the last time I thought; Is this how one should live? So I actually gave him a hug before he left … which one really shouldn’t do, but I thought: Should one live like this? (Participant nr 11)

### Limitations in Everyday Life Beyond Control

This subtheme captures how the pandemic profoundly limited participants’ everyday life, exacerbating existing vulnerabilities and resulting in experiences of boredom. The inability to meet relatives, socialize with friends, or engage in activities outside the home was experienced as particularly challenging. Shifting from physical to digital meetings, such as in churches and other associations, was not always possible due to limited technological skills or resources. Losing participation in social or activity groups also meant the loss of meaningful routines, health benefits, and intellectual engagement. As these activities were discontinued beyond the participants’ control, opportunities to maintain participation and functional abilities were limited, reinforcing the experience of restricted everyday life during the pandemic.

Relatives often constituted the primary social network; however, dependence on family members also introduced limitations in everyday life that were beyond the participants’ control. Feelings of being a burden or experiences of rejection inhibited participants from expressing their own needs, particularly during the pandemic when opportunities for alternative social interactions were limited. The inability to engage in everyday societal activities such as shopping, using public transport, strolling around town, or going out to sit on a bench was experienced as particularly restrictive.

[Talking about what the participant does when it’s no longer possible to spontaneously go out and meet people]: Shall I tell you my daily schedule? Sleep [cries] … I lie down in bed and fall asleep … it’s awful. (Participant nr 13)

I have some difficulties getting to my hairdresser; it’s hard to get it to align with home care so that they have me ready in time since they’re so very bad at planning times … they’re the worst at that … of course, Corona affects them too because they never know if they’ll have their staff there. (Participant nr 14)

### Striving for Meaningfulness in Everyday Life

A strategy used to manage the participants’ experiences of being *limited in everyday life beyond their control* was to *strive for meaningfulness.* Demonstrating resilience, the participants engaged in efforts to find meaning and continuity in their everyday life, which helped them to cope with being in double exposure. This included adapting leisure activities to fit within the restrictions, such as meeting friends outdoors, maintaining phone or digital contact, or engaging in solitary hobbies like reading, solving puzzles, or listening to the radio. Seasonal changes influenced these efforts, with socializing outdoors being more feasible in summer than in colder months.

[Talking about socializing with a neighbour]: I’ve been out quite a lot … we’ve gone out more regularly and walked … and then we’ve gone and sat down and had coffee with us … we’ve sat and talked and drank coffee and had a nice time … we’ve done this so that something happens. (Participant nr 10)

Well, one might almost say that leisure time now is TV and phone, books, walks, and newspapers … and then there’s crosswords. (Participant nr 1)

While some participants relaxed their adherence to restrictions to prioritize family interactions, others emphasized the growing importance of technology in maintaining social connections. For those with technological proficiency, efforts to learn new skills were met with varying degrees of success. Some participants expanded their use of smartphones and tablets to access social media or play games, while others relied on support from relatives for basic tasks. However, the use of digital technology was not an option for everyone, as some lacked either access to and/or competence with such technology.

Participants who resided in care homes or at home and were heavily reliant on elderly care services described unique challenges in pursuing meaningful activities. For them, the social interaction provided by care staff became increasingly important. Others sought to support peers facing greater challenges, demonstrating a sense of solidarity and shared experience.

There were two women who became very attached to me, you know… I couldn’t help it (laughs), I didn’t do anything special, but that’s how it turned out… And I supported them and so on. I did what little I could—gave them fruit, among other things, and talked with them, you know… And they passed away that same night. (Participant nr 2)

## Discussion

The present study demonstrated that the frail older people faced substantial challenges during the initial phase of the Covid-19 pandemic restrictions on societal participation, disruptions in routines, and limited capacity for independent adaptation. These conditions created what can be understood as a form of double exposure. The findings reveal how the pandemic simultaneously constrained what the older people could do, undermined the roles through which they usually expressed responsibility and identity, and weakened their sense of social connection and place. From an occupational perspective, these insights illustrate how the pandemic for the frail older people undermined opportunities for belonging, a core dimension of meaningful occupation and human well-being [24].

Participants experienced marked losses in societal participation, leading to feelings of powerlessness and limited influence over their circumstances. A novel aspect highlighted in this study was the uncertainty caused by inconsistent adherence to restrictions among elderly care staff, which further intensified participants’ vulnerability. This contributed to participants declining support for which they were eligible, despite having a clear need for it. Previous research [25, 26] in Europe similarly demonstrated that COVID-19 restrictions reduced social participation and increased vulnerability among frail older people, emphasizing the importance of a stable and predictable care environment. Further, a previous systematic review [27] highlighted the importance of professional services and social support in the care of frail older people. Elderly care staff and other care providers should have adequate competence and be able to apply professional knowledge in interactions with older people, without time constraints [28]. Together, these findings highlight the relational nature of everyday functioning in frail older people, where the ability to maintain routines and meaningful roles depends not only on individual strategies but also on environmental stability and social support [29].

Previous research [30] has demonstrated that younger older persons often deviated from public recommendations to manage essential activities during the pandemic. In the present study, those with cognitive impairment showed uneven adherence, ranging from rigid over-compliance to disregard, reflecting difficulties in interpreting and acting on public health recommendations. These contrasting patterns highlight how capability, cognitive resources, and environmental scaffolding interact to shape occupational possibilities during periods of societal disruption. At the same time, research [30–32] has shown that maintaining structure and meaning in daily life are key strategies for managing everyday pandemic-related disruptions across all ages. Consistent with this, participants in the present study described maintaining trust in society and healthcare and continued to seek necessary care when needed. High acceptance and adherence to restrictions have also been reported elsewhere [30, 31, 33].

Managing everyday life in double exposure was closely linked to experiencing limitations beyond the participants’ control in the present study. Disruptions to daily routines are known to affect activity patterns among older people [10], making the maintenance of routines central for sustaining functioning and agency. Social contact also plays a key role in cognitive ability [34], and extensive research [16, 31, 33, 35, 36] emphasizes the importance of social participation and opportunities to leave the home for mental well-being in older people. Moreover, participants in the present study sought to maintain meaningfulness in everyday life, although conscious activity selection was rarely articulated and activity adaptation was often challenging. When adaptations occurred, they typically involved increasing engagement in existing activities or strengthening established social connections. Similar patterns have been reported among Swedish older people who adapted activities to maintain engagement during the pandemic [16] and meaningful occupational engagement is known to support competence and quality of life [37]. Reduced opportunities for meaningful activities such as walking and shopping have been linked to worsening mental health among older Europeans during the pandemic [36].

The present study confirms that social isolation increased, and mental health worsened for those with already limited contacts. Similar trends have been observed among older [38] and chronically ill [32] people, where those who already were used to isolation adapted more easily and experienced fewer restrictions. Meeting relatives and friends outdoors emerged as a natural adaptation for the participants in the present study, as identified in previous studies [16, 30, 31]. Online contact represented a potential adaptation; however, its use was limited by restricted access to digital technology and cognitive impairments. Even among participants with access to such resources, support from relatives was often required. Although digital communication has been widely suggested to maintain social contacts [16, 32, 38], participants in the present study did not perceive digital interactions as replacements for in-person contact, consistent with earlier findings [16, 30, 32, 38].

Given that capability is shaped by bodily, cognitive and environmental conditions, the findings highlight the importance of person-centred approaches that align care and support with people’s wishes, needs, and priorities. Previous research has underscored the value of individual tailored, and person-centred interventions [29], and care-coordinated interventions has proven to be beneficial with regards to frailty, self-rated health [39], and performance in leisure activities [40]. Supporting capability therefore involves not only medical care but also structured efforts to maintain habits, routines and social connections that enable activity, role continuity, and belonging.

Care for frail older people should emphasize person-centred and team-based care. Such approaches are essential to meet frail older peoples’ complex physiological, psychological, and social needs through coordinated professional services [27]. Within this context, occupational therapists have a central role in identifying how frailty constrains activity performance and participation, and in supporting people to maintain meaningful routines. Interventions may include co-creating modified routines, enabling safe engagement in everyday activities, strengthening social participation through both digital and non-digital means, and working with families and care staff to stabilize expectations around daily roles. Addressing digital exclusion is particularly important, requiring both alternative means of connection and stepwise support in building digital confidence when appropriate [14]. Strengthening continuity in primary care with, for example, a designated contact person could support individualized, person-centred assessments and adaptations to increase social participation and engagement in meaningful activities [40].

Although the pandemic represented an extreme circumstance, the mechanisms identified in this study that reduced activity opportunities, disrupted roles, and weakened belonging also occur in ordinary situations where frail older people face long-term isolation due to illness, mobility limitations, or restricted social networks. Insights from this study, therefore, extend beyond the pandemic, and can inform everyday occupational therapy practice aimed at supporting frail older people whose opportunities for meaningful engagement are chronically constrained.

## Methodological Considerations

The present study had a qualitative design using semi-structured telephone interviews to collect the data. This approach allowed participants to describe their experiences in their own words while ensuring that key topics were consistently addressed. The qualitative content analysis used made it possible to explore variations in the data by identifying similarities and differences expressed in codes, categories, and themes generated from the interview material [21]. Moreover, participants were recruited through the larger RCT study [22], where older people were screened as frail when they sought emergency care. In contrast to several comparable studies [16, 30, 32–34], recruitment or participation did not at any stage occur digitally. This was considered an advantage and may have influenced the nature of participation and responses. Digital recruitment and data collection have also been noted as limitations in some of those studies [16, 30, 33].

In line with the strategic sampling used, heterogeneity was specified as an inclusion principle to ensure a broad representation of experiences among the older people. The study therefore included participants who varied in degree of frailty, age, illness, functional ability, and activity capacity. This can be seen as a strength based on the chosen method, as it provided variation in experiences of the phenomenon. No person was excluded due to age, housing situation, cognitive impairment, depression, or ability to perform daily activities, which is an additional advantage.

The interviews were conducted between September 2020 and January 2021, encompassing half a year to one year into the pandemic. When the later interviews were conducted, participants had more experience of living with restrictions compared to those interviewed initially, when the pandemic experience could still be more acute and shocking. The impact of seasons, changes in contagion levels, and the extent of restrictions during the interview period may also have affected the participants’ experiences.

All the interviews were conducted by telephone. Being interviewed by phone can give a greater sense of anonymity and security to the participants, but disadvantages include not being able to read non-verbal communication and body language [41].

Telephone interviews can sometimes be preferable to physical interviews, but the choice must always be related to the group being interviewed [42]. Given the heterogeneity among the participants, including variations in degree of frailty, cognitive abilities, and living situations, different interview formats may have been more suitable for specific participants. Some participants in this study, particularly those with higher levels of cognitive decline and degree of frailty, may have benefited from physical interviews. At the same time, other participants appeared comfortable with telephone interviews, highlighting the diversity of needs within the study group. However, due to the risk of infection during the pandemic, telephone interviews were deemed the only feasible alternative. Meeting in person could have provided more personal contact, the ability to interpret nuances and facial expressions, and opportunities to ask follow-up questions and achieve greater depth in responses. During the interviews, signs emerged that some participants had deteriorated cognitively, making it difficult to express nuances and elaborate their answers. Conducting interviews by telephone also affected participants with hearing impairments, who sometimes had difficulty perceiving what was said, requiring repetition. In some cases, participants were distracted or interrupted by events in the home environment. However, communication with participants was generally perceived to function well, and all interviews were completed and used in the study. In addition to a heterogeneous selection of participants as stated above, the collected material was assessed as sufficient to obtain a variation of experiences related to the purpose, and the results have also been verified with illustrative quotes.

To strengthen trustworthiness, the authors employed several strategies. To increase credibility, all steps in the study from design to reporting were discussed with the coauthors, who provided continuous feedback and suggestions for adjustments [23]. Overall, the present study’s methodological challenge relates to the exclusive use of telephone interviews. While this approach made data collection feasible during the pandemic and enabled participation from a broad range of people, it may also have influenced the comparability between interviews and should be considered when assessing the transferability of the findings.

## Conclusions

This study showed that frail older people lived their everyday life in double exposure during the first year of the pandemic. They experienced powerlessness under the restrictions, and limitations in everyday life beyond control, and to manage this, they accepted and trusted the imposed restrictions, alongside striving for meaningfulness in everyday life. The results suggest that frail older people may have a limited capability to adapt to new circumstances, actively make decisions to influence their situation, and proactively think about their health.

Occupational therapists have an essential role in identifying how frailty limits activity performance and participation, and in supporting the preservation or adaptation of meaningful everyday routines. These conclusions extend beyond the Covid-19 context, emphasizing the importance of society’s preparedness to support frail older people during similar event that disrupts their daily routines. Without such support, frail older people risk experiencing a life in double exposure, where both their inherent vulnerabilities and external restrictions exacerbate their challenges.

## Data Availability

No datasets were generated or analysed during the current study.
